# Practical Thoughts on Tooth Drawing

**Published:** 1853-10

**Authors:** C. T. Cushman


### *Practical Thoughts on Tooth-Drawing—Continued.—By* C. T. CUSHMAN, D. D. S.

"I am in the habit of using several different instruments suited to a variety of cases. Indeed, it cannot be questioned that the turnkey, the forceps, the elevator and the screw, are all useful in their turn. * * * * * It happens not unfrequently, that teeth are crushed and broken in the operation of extraction, even with the most skillful practitioners of our art, in consequence of the peculiar formation of the fangs or roots of the teeth, or their situation in the socket, combined with the firmness and strength of the alveoli. In these instances the removal of the roots is oftentimes attended with great difficulty."—E PARMLY.

*Difficult Case of Extraction of a Tooth.*—CASE III.—A man-servant belonging to Mr. K. had been under treatment several months for an "unmanageble head-ache," as his physician called it; was by him sent to me, with request to extract the left dens sap. of the inf. max., "if practicable."

The physician finally suspecting that this tooth (much decayed) might be instrumental in causing his headache, had attempted, a few days previously, to extract it; but only broke off the crown, considerably below the level of the gum.

There was *no portion of the roots visible* when he called on me—the gum was much swollen around them, the inflammation had also extended up the cheek. All those parts were extremely sensitive—the most careful attempt at probing around the roots, to discover their true condition, gave him intolerable pain; so much that the buccinator muscle was spasmodically affected with an incessant convulsive motion. The touch of the probe also caused a flow of blood, which instantly covered the spot. I soon satisfied myself that it was *not* practicable to extract his tooth under existing circumstances, and accordingly dismissed him.

*Two weeks after*—he called again. The inflammation had subsided considerably, although the surrounding parts were yet *tender*, and the gum bled a little at the touch of the probe. Roots not yet visible; but by forcing away the outer margin of the gum with the probe, a small portion could be seen—visible at no other point.

Without confidence of success, but simply to assure myself of the impracticability of them, I successively tried the application of the "Harris-Forceps," a slender Root-Forceps, Elevator, and Key. No one of them could secure any hold —not even sufficient to afford a *trial.* The *nips* of the first are too much curved toward each other for *such* a case, to render it at all possible to force them down to such great depth.

The Key—I have never seen one that could be made to take any hold upon a root the top of which was so far below the level of the alveolus.

The Elevator—the fracture was too low in the socket to afford any purchase. The *outer* side being the most prominent part of the fractured roots—this was barely above their fourchure. Having ascertained this by the probe, and satisfied myself of the impracticability of other methods of operating, I took my position on the left side of patient. Covering the fingers of my left hand with a napkin, I placed them within his mouth, and with the palm thus *clasped* the left side of the inferior jaw. My thumb was extended along the margin of the gum, and served as a fulcrum for the extracting instrument.

Grasping my *Punch* firmly in my right hand, I forced the point as low down as possible between the gum and root, on the *outside*, nearly perpendicularly. Keeping it firm at that point, I depressed the handle, and at the same time brought my body against it, thus raising and pushing it toward the center of the mouth.

The roots instantly gave way. I found them raised out of the socket and lying on the side. They were then easily removed by the Root-Forceps.

The patient was overjoyed; declared it didn't hurt him, and that he would pay me himself if he were able. The fracture of the crown had extended on the inside to the *fourchure* of the roots; on the anterior and posterior sides equally low; outside a little above—barely substance left to keep them together during the operation.

The roots were long, bifurcated, and measured together much wider across the apices than at the base. The periosteum was extensively ulcerated, and I have no doubt, contributed principally to his headache during the many months of his suffering. I am at a loss to conceive in what other practicable way these fractured roots could have been extracted.

By this method of using the Punch, I have but rarely failed in such cases; and never found it necessary to cut away the alveolus, as is done for the application of a different instrument.

I object to this operation where it can be dispensed with—not being content with the assurance that if not cut or broken away the bone will become *absorbed* away just the same. I believe the collapse must be greater than after ordinary extractions; for in many mouths we see the gum fully *rounded* over a vacant socket, as though the roots were yet within it Not so after the *cutting* operation. Besides, it is a longer, and more painful method.

*Unlooked-for Difficulties in Extraction of Teeth.* Case IV.—Oct. 3d, 1851.—A stout negro man, with full denture, teeth hard and firm-set—the first left inferior molar been aching a long time—a case of violent Periostitis; tooth sore and gum inflamed around with fistula on outside.

Tooth *appeared* a little loose and gave promise of extraction with but little difficulty or pain. (I had extracted its *mate* opposite, a year before, without difficulty). This had a large cavity on its posterio-lingual and rinding surface, which opened into the root. I scarified and applied the Harris Forceps,* forcing it *as low down as possible.* The crown and anterior root only came away, leaving the posterior one broken off at the *fourchure.* The tooth was decayed so low as almost to separate the roots.

The remaining root gave me immense trouble to remove. It could not be seized by any Forceps. The Elevator could not be made to secure a hold between it and the 2d molar. It was exceedingly sensitive to the touch, and probing gave great pain. The bleeding was profuse. I started it with the *Punch*—standing on the left side, and operating as in the preceding case.

The periosteum was in the highest possible state of inflammation. A large ulcerous sac was attached to the apex of the root.

*Case V.*—Mrs. A. æt. 30, Nervo-lymphatic—inferior jaw full denture—2d molor left side been aching—mostly at night—for a week—face a little swollen. Tooth had large cavity in posterio-bucеal and grinding surface, which, as could be plainly seen, opened into the root. The tooth had a bluish necrosed appearance throughout. A plain case of Periostitis—tooth sore to the touch and all the neighboring teeth sore. It gave accordingly promise of being extracted easily—with but little force. Patient seated in a low chair, with head supported. Scarified and applied the *Harris Forceps* as low down on the tooth as possible. *I could scarcely move the tooth in its socket.* Finally it raised up a little and appeared to be *working out* slowly. The crown with about *half* the length of the roots came away, presenting a smooth level fracture across both, about one line below the point of seizure—the remainder of the roots were left, as firmly articulated as before.

---

* I know of no better instrument, for general use, for all the inferior molars, particularly of the left side. For the 1st molar of the RIGHT side, it brings the hand and forearm rather too far in advance of the body, which occasions a loss of power. For this particular tooth, the HAWKSBILL Forceps is perhaps preferable.

The *only* chance (and that did not appear very promising) now seemed to be with the *Punch.* I applied it precisely as in Case 3d, and successfully, at the first effort, dislodged the *posterior* root.

She flung it into the door-yard before I saw it. Examination now showed the anterior root to be loosened—this I brought out at first trial with the simple *hook,* (Thumb Fulcrum) held in the hand somewhat like a pestle, and applied to the lingual side of the root.

The periosteum was in precisely the same condition, as that of Case 3d and had a large sac. She did not complain at all during the operation, and assured me that "it did not hurt much."

*Moral.*—One should not promise easy success in extracting *any* tooth—no matter how easy it *looks* to be.

The Punch and Thumb-Fulcrum are valuable—sometimes indispensable instruments, for the extraction of roots of teeth.

*A Fixed Fact.*—CASE VI.—A middle aged gentleman, cf bilious temperament, had a superior 2d molar decayed on the poserior surface ; and in order to have it *plugged*—"to get at it"—his dentist advised the extraction of the contiguous *dens sapientia,* which was sound. He gave "*him three fair and separate trials* at it, and he never budged it."

It was then abandoned ; and when I saw it, nearly a year subsequently, it remained in *statu quo.*

In connection with the preceding, as further illustrating and confirming the fact that the teeth of *all the members of certain families are articulated with an extraordinary degree of firmness,* I will relate the case of a son of the foregoing.

*Extraordinary Difficulty in Extracting a Tooth that promised easy success.*—CASE VII.—Master J., æt 14, Nervous bilious temperament, (Oct. 21, 1851), his 2d left inferior bicuspid to be extracted. *All* the inferior teeth standing —including the 2d molars—close, but not crowded.

Seated in the chair, with low seat ; I stood over him on the left side and applied the lower Incisor and Bicuspid Forceps, at the same time grasping the chin and jaw in my left hand. A pull sufficient to extract such a tooth in an adult—any ordinary case—scarcely loosened it ! I rested and took another, which raised it about the sixteenth of an inch, when it was wedged or *bound*, it seemed firmer than it was primarily !

A third pull worked it a little higher, but left it still *fast.* The *fourth* brought it out, and revealed a root of *extraordinary* length, breadth and thickness. A quarter of an inch from the apex it bent posteriorly at a considerable angle

This it was probably, which *bound* or "jammed" against the alveolus of the molar. It was a case that would have tested severely the virtue of *ligament cutting*, and power of extracting with *thumb and finger*; and would have even proved fatal to the pretension of him who boasted that he always extracted teeth without pain or difficulty

*A Stumper for the Dentists.*—CASE VIII.—Dr. —— with firm-set, regular, and generally sound teeth; showed me his right inferior *dens sap.* growing in such a position as to be completely *dovetailed* behind the 2d molar

Not perceiving any cause for this deviation in growth, it yet leans forward at such an angle that the anterior prominence of the crown, on the lingual side, is barely on a level with the gum ; on the buceal side it is a little higher; it therefore leans slightly inward.

The posterior prominence is about on a level with the surface of the other molars. The pressure exerted by this *growing* tooth (attempting to grow) seems to be very great; he experiences at times a severe "grinding" pain in the 2d molar, which is only slightiy defective—as is the 1st, on its contiguous surface. Both, however, could be permanently cured by plugging. The irregulartooth is only slightly decayed on the anterior part of its grinding surface—the cavity being of course almost under the 2d molar. He has had trials of extraction os this irregular tooth by two persons. The first made painful efforts to introduce the *Elevator* between it and the 2d molar; but this is utterly impossible to accomplish. Trials were also made with the Forceps; but any amount of force exerted in the usual manner, would only be expended upon the 2d molar, under which it is firmly *locked*. It was then abandoned. On applying to the second professional, he gave ample assurance of extracting it *extempore*. But alas! for human infallibility. Various and repeated projects in sapping and mining resulted only in pain insufferable to the patient, and discomfiture to the engineer. The enemy wasn't "routed;" but simply remained *rooted* to the spot. An adjournment was moved and carried *sine die*. The tooth appears exceedingly firm; all the teeth are firmly articulated. I made no attempt upon it: were I to do so, I can conceive of no more feasible plan than the use of the *Physic-Forceps*. With this instrument I believe it might be extracted. It must certainly be forced in a *backward* direction, at least a line, before it can clear the 2d molar, so as to admit of its being *raised* from its socket.

